# Repeatability of Freehand Implantations Supported with Universal Plastic Sleeves—In Vitro Study

**DOI:** 10.3390/ijerph17124453

**Published:** 2020-06-21

**Authors:** Łukasz Zadrożny, Marta Czajkowska, Eitan Mijiritsky, Leopold Wagner

**Affiliations:** 1 Department of Dental Propaedeutics and Prophylaxis, Medical University of Warsaw, 02-006 Warsaw, Poland; zpips@wum.edu.pl (Ł.Z.); lwagner@wum.edu.pl (L.W.); 2 Department of Laryngology, Medical University of Silesia, 40-027 Katowice, Poland; 3 Department of Otolaryngology, Head and Neck and Maxillofacial Surgery, Tel-Aviv Sourasky Medical Center, Sackler Faculty of Medicine, Tel Aviv 6139001, Israel; mijiritsky@bezeqint.net; 4 The Maurice and Gabriela Goldschleger School of Dental Medicine, Tel Aviv University, Tel Aviv 6997801, Israel

**Keywords:** implantology, precision, repeatability

## Abstract

Repeatability and precision are major factors which have an influence on final implant treatment results. The aim of this study was to evaluate the repeatability of freehand implant procedures assisted with special plastic sleeves which are placed on the drill to guarantee the proper mesiodistal distance from the landmark’s surface. Patient data required for implant treatment, including images of mandibles from CBCT scans and virtual models of soft tissues and teeth, were used to prepare complete virtual models of patient clinical conditions. The models were saved as STL files and 3D printed in five copies. Drilling procedures were done at positions 37, 46 and 47. Each model was scanned with pins in the osteotomies and compared using digital aligning of the models’ surfaces. The average deviation was −1.38 ± 1.4 mm. Average deviations on guide pins placed at position 37 were −0.46 ± 0.59 mm, at position 46 were −1.46 ± 0.88 mm (landmark’s surface of both: distal tooth’s surface), and at position 47 were the highest: −2.69 ± 1.62 mm (landmark’s surface: surface of plastic sleeve). The following conclusion was drawn: Using universal plastic sleeves could improve implant procedure precision especially in the case of partially edentulous patients.

## 1. Introduction

Repeatability and precision are major factors which influence final implant treatment results. There is no doubt that clinicians should plan implant procedures based on careful clinical examination and analysis of CBCT (Cone beam computed tomography) scans to choose the best treatment option [1].

However, in some cases, inaccuracies are introduced while transferring a virtual treatment plan to the patient’s mouth. Several different kinds of guide templates have been introduced to provide proper positioning and angulation of implants in the bone [2]. Implant placement in respect of the prosthodontic plan plays a crucial role in the long-term predictability and success of implant prosthetics. Moreover, a prosthetically-driven implant placement accomplished with a surgical guide can decrease clinical and laboratory complications. Increase of demand for dental implants has resulted in the development of state-of-the-art techniques for the fabrication of these templates [1].

A number of studies show that users of CAD/CAM (Computer-aided design/Computer-aided manufacturing) surgical guides may experience problems with accuracy and repeatability of dental implantation procedures [2,3,4,5,6,7,8]. In the literature we can also find descriptions of a few problems with clinical application of guides (e.g., the guide fixation, its stability and limited mouth opening) [9,10]. Hence several surgical kits were introduced to help clinicians with proper implant positioning when a digital guide is not available.

The aim of this study was to evaluate the repeatability of freehand implant procedures assisted with special plastic sleeves which are placed on implant drills to assure the proper mesiodistal distance from patient teeth or anteriorly prepared implant sites. 

## 2. Materials and Methods 

**Figure 1:**
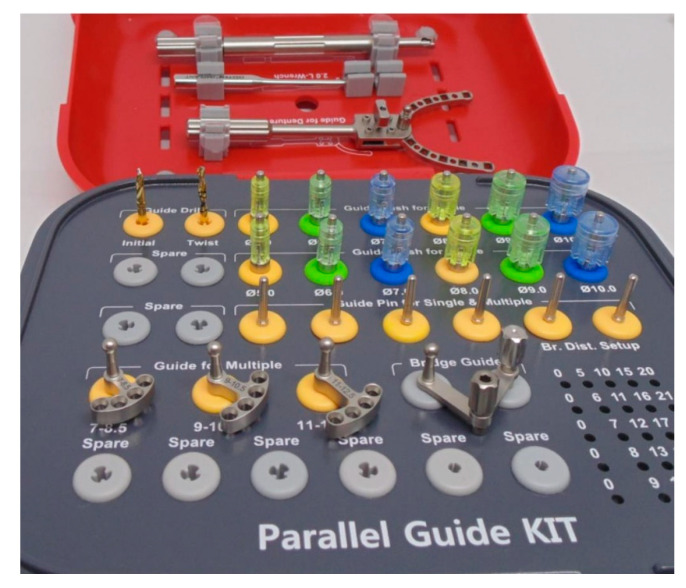
Kit includes: 2 twist drills, plastic sleeves in diameters 6 to 10 mm corresponding to the mesiodistal dimensions of different teeth, fixed parallel distancers from 7 to 12.5 mm, adjustable distancer for bridges, adjustable guide for edentulous ridges, parallel pins, and holders.

The following experiment was planned to check repeatability of implant procedures with a kit that includes plastic sleeves of different diameters corresponding to the mesiodistal dimensions of different teeth (Parallel Guide Kit, Osstem Implants, Seoul, Korea) (Figure 1).

Patient data required for implant treatment, like an image of the mandible from a CBCT scan and a virtual model of soft tissues and teeth, were used to prepare a complete virtual model of patients’ clinical conditions. For this purpose, DDS-Pro software (JST, Częstochowa, Poland) for virtual treatment planning was used. The same software was used for supporting the diagnosis process and for choosing the best place for implantation. The model was saved as an STL file and 3D printed in five copies in a SONDASYS SL01 device using Selective Laser Sintering (SLS) technology (material: polyamide powder with aluminum nanofiller and fiberglass, Sondasys, Ogrodzieniec, Poland). Drilling procedures were done with a Parallel Guide Kit. Positions of implants were chosen to be placed with the use of 10 mm diameter sleeves for each implant. The sleeves touched the distal surface of tooth 36 at position 37, the distal surface of tooth 45 at position 46, and the 10 mm sleeve already placed on a guide pin at tooth 46 in position 47.

After preparing osteotomies, guide pins were placed and each model was scanned with an optical scanner (Cs3600, Carestream, Atlanta, GA, USA) to create a 3D model in the STL file. Each one of 5 STL files was then compared with four others using free software HP 3D Scan 5 (HP, Palo Alto, CA, USA). The method of measurement is shown in Figure 1.

**Figure 2:**
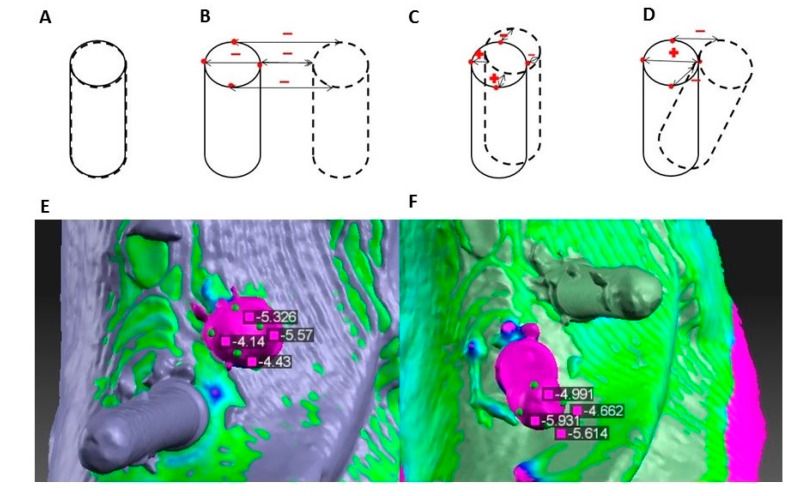
The method of measurement. The continuous line represents the reference model and the dotted line represents the test model. Reference points are marked by red dots. (**A**) Guide pins on both models are in the same place—deviations are equal to 0 mm. (**B**) Guide pin on the test model is implemented beside the guide pin on the reference model—all deviations on reference points are minus. C. and D. Guide pin on the test model is slightly moved (**C**) or implantation angle is different (**D**)—deviations on the reference points are plus or minus depending on whether the nearest surface of the test model is covered by the reference model or not. (**E**) Deviation on model number 4 in comparison with model number 5 (5 vs. 4). (**F**) Deviation on model number 5 in comparison with model number 4 (4 vs. 5).

After preparing the implant sites, guide pins were placed and each model was scanned with an optical scanner (Cs3600, Carestream) to create a 3D model as an STL file. Each one of 5 STL files was then compared with four others using HP 3D Scan 5 software (HP, Palo Alto, CA, USA). The method of measurement is shown in Figure 2.

STL files show the object surface as a set of triangles. Surfaces of all five models were almost the same outside of the place of the guide pins. Because of this, it was possible to align scans with each other. On the model which served as a reference in this measurement, four reference points were marked on each of the guide pin tops based on their geometric structure. After comparing, surface software showed values of deviation in each point: pm—mesial point, pd—distal point, pl—lingual point, pb—buccal point. In each of the series of measurements these points were marked only once, so when test models were changed, they stayed in exactly the same place on the reference model. Thus, benchmarks for assessing similarity of particular samples were the same.

Sixty measurements were made in all—each of the five models with 3 guide pins was both a test and a reference. Figure 2 also shows how the deviation values changed depending on which model was the reference one.

Values of deviation on all points were recorded for each guide pin for each measurement. All data was reported as the mean ± standard deviation. Correlations between implant placement and level of average deviation on guide pins were analyzed by chi square test. Statistical analyses were performed using Microsoft Office Excel for Windows (Microsoft 2018, Redmond, WA, USA). A *p*-palue < 0.01 was considered significant.

## 3. Results

**Figure 3:**
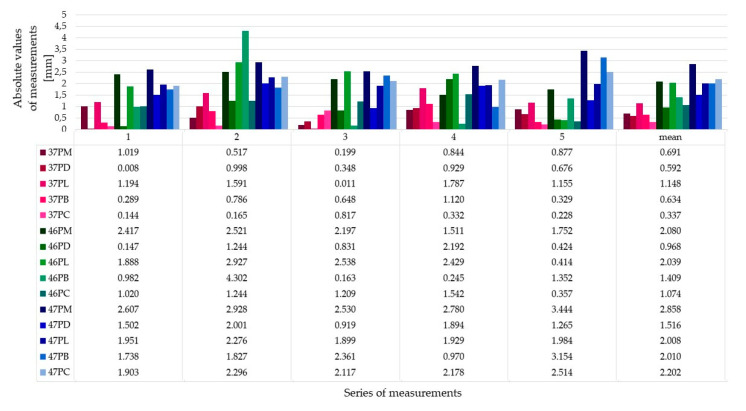
Absolute values of measurements which were done on each of five measurements points on each insert in positions 37, 46 and 4. Name of measurement point: tooth number and abbreviation of “Point Name”: PM—point mesial, PD—point distal, PL—point lingual, PB—point buccal, PC—point central.

Figure 3 shows the absolute values of measurements (mm) in each of the measurement series. This figure is an overview of our results.

**Figure 4:**
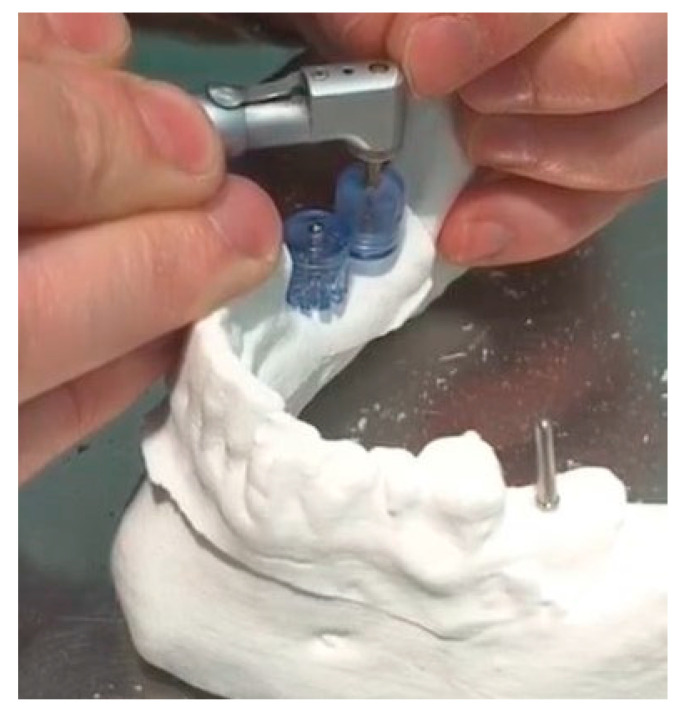
Drilling at site 47 with reference point of sleeve at pin in site 46.

**Figure 5:**
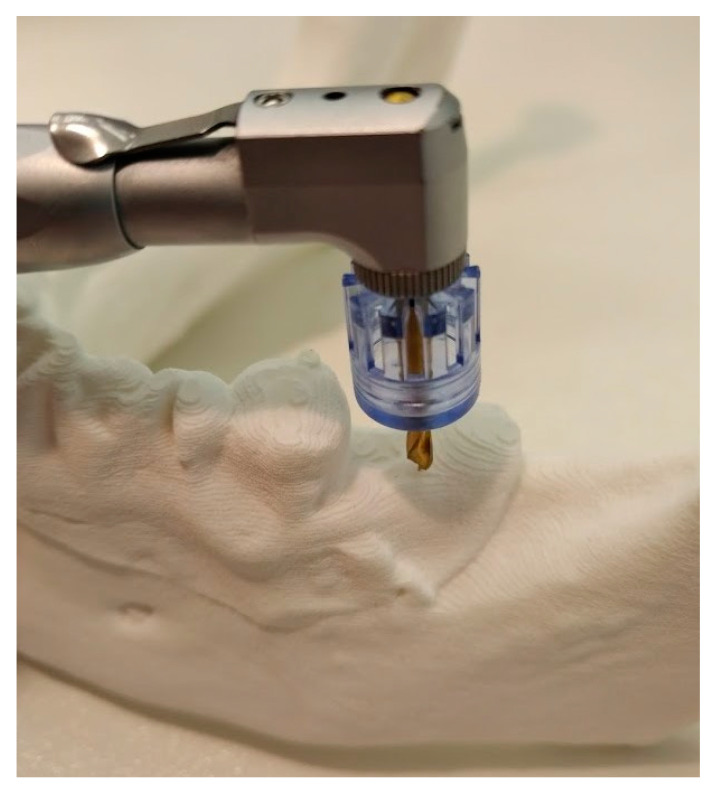
Drilling at site 37 with reference point of distal surface of tooth 36.

The average deviation was −1.38 ± 1.4 mm. The differentiation of deviation values depended on implant placement. The standard deviation value indicated that the differentiation of samples was high, despite the fact that average deviation was low. This observation suggested that there is a correlation between the benchmark for implantation and its repeatability. Average deviation on the guide pin placed at position 37 was −0.46 ± 0.59 mm, and at position 46 it was −1.46 ± 0.88 mm. During implantation of these 10 implants, the plastic sleeve touched the previous tooth. Implant sites at position 47 were prepared with another 10mm sleeve touching the 10 mm sleeve on the pin guide at position 46. The average deviation in this group of five guide pins was the highest: −2.69 ± 1.62 mm. Analyzing the results one has to consider that the crest of the ridge in areas 46 and 47 was tilted buccally compared to the more even and flat crest at site 36 (Figure 4 and Figure 5).

**Figure 6:**
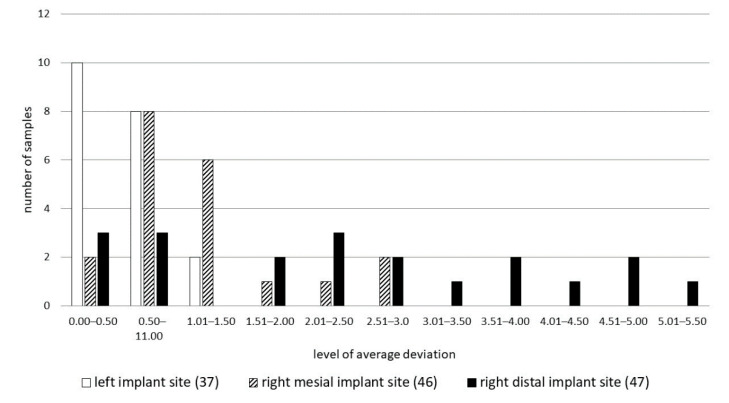
Number of samples in which average deviation (AD) is in the specified level of average deviation.

Figure 6 presents the results of a chi square test. All twenty samples are matched to one of the levels of possible average deviation. The results of the chi square test allow us to reject the hypothesis about quality independence. As we can see on the bar chart only right mesial implants have the highest levels of average deviation. This group was also characterized by the highest differentiation.

## 4. Discussion

In vitro studies provide stable conditions during series of procedures. Three-dimensional printed models properly represent the shape of the alveolar bone. The authors must mention that a limitation of this study is that the material which was used for model manufacturing was harder than bone, which caused initial difficulties with drilling. The material which was cut during implant site preparation was depositing on the drill, making it difficult to perform full-depth insertion. However, these models and digital methods of sample comparison contribute to eliminating invasive procedures and increasing objectivity and precision of measurements.

The main purpose of this examination was to evaluate repeatability of freehand implant procedures assisted with special plastic sleeves. The results of this study show that using such support could help improve the precision of freehand implant surgery in the case of replacing a single missing tooth but is not as good as using individual implant guides. 

In the research by Vermeulen et al., where the accuracy of implant placement with and without using implant guide templates was examined, it was shown that average deviation for placement with a guide was 0.42 ± 0.52 mm and without a guide was 1.27 ± 1.28 mm [11]. As we can see, the average deviation of freehand implant placement without any assistance is even slightly lower than with the support of plastic sleeves. This incoherence could be caused by different methods of measurement and sample size. 

The meta-analysis of Tahmaseb et al., based on 20 clinical studies including placing 2238 implants using static guides in 471 patients, revealed a total mean error of 1.2 mm (1.04 mm to 1.44 mm) at the entry point, 1.4 mm (1.28 mm to 1.58 mm) at the apical point and deviation of 3.5° (3.0° to 3.96°). This meta-analysis, as well as the present study, both emphasize statistical significance differences in accuracy in favor of partial edentulous compared to full edentulous cases [12].

Analysis of in vitro studies and systematic review, as well as multicenter clinical trials and randomized controlled trials, lead to the conclusion that using individual guides may be the best way to achieve elevated accuracy and remain in line with the treatment plan [7,8,10,11,12,13,14]. However, limited surgical access especially in the distal area of the alveolar ridge with surgical guides intraorally, may be one of the most common challenges in using surgical guides [4,14]. Tallarico et al. found that even if the accuracy of guided surgeries in the anterior region was more than good, accuracy dropped in the distal area where some guide movement may occur, or open sleeve design has to be applied. However, the same author in another study comparing guided and freehand approaches achieved successful results over the five-year follow-up period for both techniques. Clinically we have to evaluate all advantages and limitations of guided implant surgeries, such as surgical time, level of post-operative pain and swelling, and overall costs and time required to perform a fully guided surgery [2,14,15]. Proper and careful diagnosis is the basis of present-day implantology. The whole process of preparing digital implant templates forces clinicians to carefully analyze CBCT scans as well as intraoral scans, or stone or virtual models and leads to careful planning of each specific case [16]. Considering individuals’ needs as well as clinical limitations including surgical access and guide fixation, as well as costs required to produce the guide, clinicians may decide to use simpler and less expensive tools to transfer the digital plan to the patient’s mouth. 

## 5. Conclusions

Within the limitations of this in vitro study, the following conclusion was drawn: using universal plastic sleeves could improve implant procedure precision over a freehand approach especially in the case of partially edentulous patients. The authors believe that clinical scientific research should focus more in identifying which clinical situations can get the greatest benefits from implant guided surgery and which interventions may be performed successfully, faster or more cheaply with the use of different tools, e.g., the kit used in this study. 

Regarding further studies by means of 3D printed models, there is a need to analyze which 3D printing material will be most appropriate in terms of consistency, strength and hardness. Furthermore, there is still a need for more studies such as randomized controlled trials that comprehensively assess the advantages and disadvantages of fully digital surgical protocols in specific clinical situations.
